# Ross River Virus Antibody Prevalence, Fiji Islands, 2013–2015

**DOI:** 10.3201/eid2504.180694

**Published:** 2019-04

**Authors:** Maite Aubry, Mike Kama, Jessica Vanhomwegen, Anita Teissier, Teheipuaura Mariteragi-Helle, Stephane Hue, Martin L. Hibberd, Jean-Claude Manuguerra, Ketan Christi, Conall H. Watson, Eric J. Nilles, Colleen L. Lau, John Aaskov, Didier Musso, Adam J. Kucharski, Van-Mai Cao-Lormeau

**Affiliations:** Institut Louis Malardé, Papeete, French Polynesia (M. Aubry, A. Teissier, T. Mariteragi-Helle, D. Musso, V.-M. Cao-Lormeau);; Fiji Centre for Communicable Disease Control, Suva, Fiji (M. Kama);; The University of the South Pacific, Suva (M. Kama, K. Christi);; Institut Pasteur, Paris, France (J. Vanhomwegen, J.-C. Manuguerra);; London School of Hygiene and Tropical Medicine, London, UK (S. Hue, M.L. Hibberd, C.H. Watson, A.J. Kucharski);; World Health Organization Division of Pacific Technical Support, Suva (E.J. Nilles);; Harvard Medical School and Brigham and Women’s Hospital, Boston, Massachusetts, USA (E.J. Nilles);; Harvard Humanitarian Initiative, Cambridge, Massachusetts (E.J. Nilles);; Queensland University of Technology, Brisbane, Queensland, Australia (J. Aaskov);; Australian National University, Canberra, Australian Capital Territory, Australia (C.L. Lau);; Aix Marseille Université, IRD, AP-HM, SSA, VITROME, IHU-Méditerranée Infection, Marseille, France (D. Musso)

**Keywords:** Ross River, arbovirus, seroprevalence, immunoglobulin G, Fiji, Pacific, viruses, mosquitoborne diseases, vector-borne infections

## Abstract

A unique outbreak of Ross River virus (RRV) infection was reported in Fiji in 1979. In 2013, RRV seroprevalence among residents was 46.5% (362/778). Of the residents who were seronegative in 2013 and retested in 2015, 10.9% (21/192) had seroconverted to RRV, suggesting ongoing endemic circulation of RRV in Fiji.

Ross River virus (RRV) is an *Alphavirus* of the family *Togaviridae* and is transmitted to humans by *Aedes* and *Culex* mosquitoes (*1*). Marsupials are considered important reservoirs of RRV (*1*). Clinical manifestations of RRV infections include fever, rash, and arthralgia. RRV is endemic to Australia, where it causes ≈5,000 cases of epidemic polyarthritis annually (*1*). Outbreaks of RRV infection were reported during 1979–1980 in Fiji, the Cook Islands, American Samoa, New Caledonia, and Wallis and Futuna (*1*–*3*). Subsequently, RRV infections were detected in travelers returning from Fiji during 1997–2009 (*3*) and in patients with suspected dengue in Fiji in 2005 (*4*).

The Republic of the Fiji Islands comprises 322 islands distributed among 4 administrative divisions (Central, Western, Eastern, and Northern) and has a population of ≈830,000. Apart from RRV, the 4 serotypes of dengue virus were the only mosquitoborne viruses known to circulate in Fiji until the recent emergence of Zika and chikungunya viruses (*5*). We report evidence of endemic RRV circulation in Fiji on the basis of serologic analysis of blood samples collected in 2013 and 2015.

Our study included 778 participants recruited during September–November 2013 from the Central, Western, and Northern divisions for a community-based serosurvey for leptospirosis and typhoid (*6*). Among the residents from the Central division who had participated in the 2013 survey, 333 had blood drawn again during October–November 2015, including 311 whose serum sample collected in 2013 was available for testing. We tested all blood samples for RRV IgG by using a recombinant antigen-based microsphere immunoassay (*7*). We analyzed the data with GraphPad Prism 6.03 using the Fisher or χ^2^ test. We considered p values <0.05 to be significant. 

The prevalence of RRV antibodies among participants in 2013 was 46.5% and was lower in the Central (38.1%) than the Western (58.6%; p<0.0001) and Northern (55.9%; p = 0.0108) divisions (Table). The prevalence of RRV antibodies among the participants sampled in the Central division in 2015 (37.2%) was similar to results from this division in 2013 (38.1%). In 2013, a total of 37.4% of the participants born after 1982 (postoutbreak) had RRV antibodies, and this rate in 2015 (26.9%) was not significantly different (p = 0.0685). The prevalence of RRV antibodies increased with age (p<0.0001 in 2013, p = 0.0020 in 2015) and was higher in rural than in urban (p<0.0001 in 2013, p = 0.0197 in 2015) and periurban areas (p = 0.0060 in 2013). No difference by sex was observed. Among the 311 participants with available serum samples collected in both 2013 and 2015, a total of 21 (10.9%) of the 192 participants who had no detectable RRV antibodies in 2013 had seroconverted to RRV by 2015 (data not shown).

**Table:** Prevalence of Ross River virus antibodies in a representative subset of the population of Fiji sampled during September–November 2013 (n = 778) and October–November 2015 (n = 333)*

Variable	No. seropositive/mo. tested (% [95% CI])	
	2013	2015
Birth year		
<1982	197/336 (58.6 [53.4–63.9])	68/144 (47.2 [39.6–55.7])
>1982	165/441 (37.4 [32.9–42.1])	56/189 (29.6 [23.2–36.7])
1982–1990	58/117 (49.6 [41.1–58.9])	17/38 (44.7 [31.4–61.4)
1991–2000	66/146 (45.2 [37.7–53.6])	20/66 (30.3 [21.3–42.9])
2001–2010	40/170 (23.5 [18.1–30.7])	19/83 (22.9 [15.8–33.5])
>2011	1/8 (12.5 [0.3–52.7])	0/2 (0.0 [0.0–84.2])
Sex		
F	195/423 (46.1 [41.5–51.0])	73/190 (38.4 [32.1–45.8])
M	167/354 (47.5 [42.2–52.5])	51/143 (35.7 [28.6–44.1])
Division		
Central	172/451 (38.1 [33.9–42.8])	124/333 (37.2 [32.4–42.7])
Northern	33/59 (55.9 [44.1–68.7])	ND
Western	157/268 (58.6 [52.8–64.5])	ND
Zone		
Rural	189/344 (54.9 [49.8–60.3])	52/113 (46.0 [37.5–55.6])
Periurban	55/135 (40.7 [33.2–49.5])	27/77 (35.1 [26.0–46.8])
Urban	117/298 (39.3 [34.1–45.1])	45/143 (31.5 [24.8–39.8])
Total	362/778† (46.5 [43.1–50.1])	124/333 (37.2 [32.4–42.7])

*ND, no data (participants were not recruited from the Northern and Western divisions in 2015). †For 1 participant, information about age and sex were not available; for another participant, information about the zone of residence was not available.

A serosurvey conducted after the RRV outbreak in Fiji in 1979 detected RRV antibodies in 92% of the participants from the Western division (*2*). In our study, which was conducted in 2013, RRV antibody prevalence in the Western, Central, and Northern divisions ranged from 38.1% to 58.6%, and 37.4% of persons born after 1982 had RRV antibodies, suggesting that RRV circulated in all 3 divisions after the 1979 outbreak. The report of RRV infection in travelers or inhabitants from Fiji during 1997–2009 (*3*,*4*), the observations that 10.9% of the seronegative participants in our study seroconverted to RRV during 2013–2015, and the increase in the prevalence of RRV antibodies with age, strongly suggest endemic RRV transmission in Fiji.

The finding that RRV seroprevalence was higher in rural than in urban and periurban environments suggests increased transmission risks in the rural areas, potentially because of higher-risk occupations of rural residents (including farming and outdoor work), greater exposure related to rural housing or other environmental factors, greater animal reservoir density, the possibility that nondomestic mosquito species in Fiji such as *Aedes vigilax*, *Ae. polynesiensis*, *Ae. pseudoscutellaris*, *Ae. albopictus*, and *Culex annulirostris* might be more competent vectors of RRV than peridomestic mosquito species such as *Ae. aegypti* (*8*).

Serosurveys conducted in American Samoa in 2010 (*1*), in French Polynesia during 2011–2013 (*9*) and 2014–2015 (*7*), and our study in Fiji during 2013–2015 suggest that endemic circulation of RRV in the Pacific region continued, or recommenced, after 1979–1980. These data provide further evidence for endemic transmission of RRV in areas where marsupials are absent (*10*). Because of extensive travel between Australia and the Pacific Islands, it is plausible that RRV is repeatedly seeded into the Pacific region. Whether this plays an important role in perpetuating local transmission in the Pacific Islands is unknown. As previously illustrated with Zika and chikungunya viruses, a risk exists for emerging arboviruses to be imported from the Pacific to other parts of the world, and RRV could be the next unexpected threat.
